# Prevention of Incident Hypertension in Patients With Obstructive Sleep Apnea Treated With Uvulopalatopharyngoplasty or Continuous Positive Airway Pressure: A Cohort Study

**DOI:** 10.3389/fsurg.2022.818591

**Published:** 2022-03-24

**Authors:** Yi-Chih Lin, Chun-Tien Chen, Pin-Zhir Chao, Po-Yueh Chen, Wen-Te Liu, Sheng-Teng Tsao, Sheng-Feng Lin, Chyi-Huey Bai

**Affiliations:** ^1^Department of Otolaryngology, Shuang Ho Hospital, Taipei Medical University, New Taipei City, Taiwan; ^2^Sleep Center, Shuang Ho Hospital, Taipei Medical University, New Taipei City, Taiwan; ^3^School of Public Health, College of Public Health, Taipei Medical University, Taipei, Taiwan; ^4^Department of Otolaryngology, School of Medicine, College of Medicine, Taipei Medical University, Taipei, Taiwan; ^5^Department of Otolaryngology, Wan Fang Hospital, Taipei Medical University, Taipei, Taiwan; ^6^Department of Chest, Shuang Ho Hospital, Taipei Medical University, New Taipei City, Taiwan; ^7^Department of Public Health, School of Medicine, College of Medicine, Taipei Medical University, Taipei, Taiwan; ^8^Department of Emergency Medicine, Taipei Medical University Hospital, Taipei, Taiwan; ^9^Nutrition Research Center, Taipei Medical University Hospital, Taipei, Taiwan

**Keywords:** hypertension, obstructive sleep apnea, uvulopalatopharyngoplasty, sleep surgery, continuous positive airway pressure

## Abstract

**Purpose:**

To determine whether treatment with uvulopalatopharyngoplasty (UPPP) or continuous positive airway pressure (CPAP) in patients with obstructive sleep apnea (OSA) prevents hypertension, compared to those not receiving any treatment.

**Methods:**

A retrospective cohort study was conducted among 413 patients with OSA (age ≥ 35 years) at the Shuang Ho Hospital between 2009 and 2016. The patients were divided into three groups: UPPP, CPAP, and non-treatment groups. Data about the personal characteristics, history of comorbidities, and polysomnography (PSG) reports were collected at baseline. A Cox model with inverse probability of treatment weighting was used to adjust for confounders and baseline diversity.

**Results:**

After multivariate adjustment and weighting for incident hypertension, patients in both the CPAP and UPPP groups showed a significant preventive effect on hypertension than in the non-treatment group. Moreover, patients in the CPAP group had lower event rates than those in the UPPP group.

**Conclusion:**

UPPP can prevent the development of new-onset hypertension in patients with OSA. CPAP had a better preventive effect than UPPP. UPPP might be a good alternative for reducing the risk of the onset of hypertension when compliance to CPAP is poor.

## Introduction

Obstructive sleep apnea (OSA), the most common sleep-related breathing disorder, has an enormous burden on global health and the economy. The worldwide prevalence is 9–38% (1). Advancing age, male sex, and higher body mass index (BMI) are the three main factors that increase the prevalence of OSA (1, 2). These three factors are also highly related to hypertension (3–5).

OSA is characterized by repeated upper airway collapse, which causes intermittent hypoxemia and sleep fragmentation (6). These effects of OSA might contribute to increased blood pressure (7). International guidelines recognize OSA as one of the most common risk factors for resistant hypertension (8). In a cross-sectional study, patients with resistant hypertension had a high percentage (>70%) of OSA (9). The Wisconsin Sleep Cohort Study showed that patients with moderate to severe OSA [apnea-hypopnea index (AHI) score ≥ 15/h] had a 3.2-times higher risk of incident hypertension than patients without OSA (7).

Continuous positive airway pressure (CPAP) therapy was found to reduce the blood pressure in randomized control trials among patients with OSA accompanied by hypertension (10, 11). It was found to decrease the 24-h mean and diastolic blood pressure in patients of OSA with resistant hypertension (12). A previous meta-analysis reported that CPAP therapy led to a 13.3 mmHg reduction in pulmonary artery pressure among patients with OSA (13), which means hypoxic vasoconstriction, vascular remodeling, and endothelial dysfunction increased the risk of hypertension. A prospective cohort study showed that CPAP therapy could lower the risk of incident hypertension (14). However, another randomized controlled trial showed that CPAP therapy could not decrease the incidence of hypertension in non-sleepy patients with obstructive sleep apnea (15).

Uvulopalatopharyngoplasty (UPPP), one of the alternatives to CPAP, has been shown to reduce the AHI score in patients with OSA (16). One trial involving 65 patients with OSA demonstrated Ssignificantly reduced blood pressure after UPPP (17). However, only a few studies have shown that UPPP can decrease blood pressure in patients with OSA and hypertension.

CPAP therapy acts as a pneumatic splint to maintain the upper airway patency during sleep, preventing the soft tissues from collapsing. UPPP can increase the retro-palatal lumen and decrease the collapsibility of the pharynx. Both treatments can decrease episodes of hypopnea or apnea and finally prevent an increase in blood pressure. Therefore, treatment of OSA might help in the development of new-onset hypertension in OSA.

The primary preventive effect of UPPP on hypertension is unclear, and related studies are still limited. CPAP therapy is highly efficacious in preventing the risk of OSA; however, its use has limitations in the real world. This study aimed to determine whether UPPP and CPAP in patients with OSA above 35 years had a preventive effect on the occurrence of hypertension.

## Methods

### Study Sample

**Figure 1:**
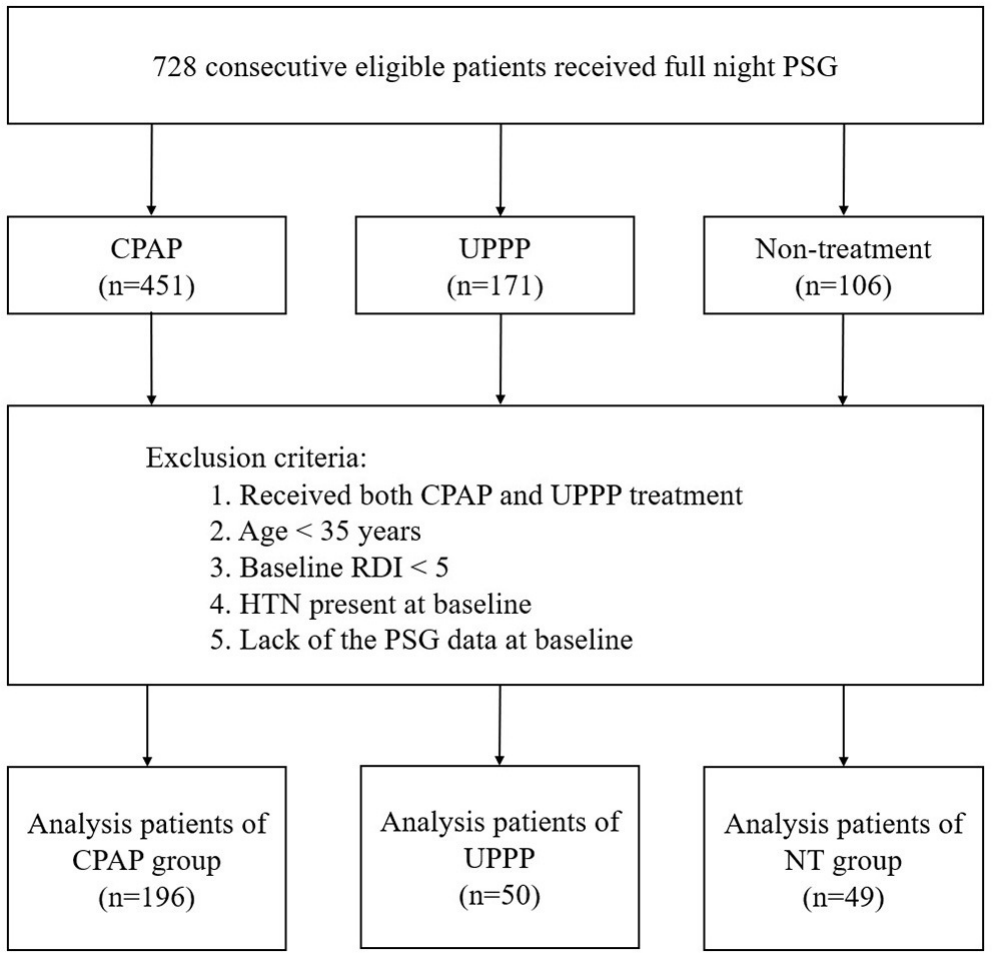
Enrollment of the study patients. CPAP, continuous positive airway pressure; UPPP, uvulopalatopharyngoplasty; RDI, respiratory disturbance index; OSA, obstructive sleep apnea; NT, non-treatment.

This was a retrospective cohort study of 622 patients treated for OSA and 106 patients with OSA who were not treated at Shuang Ho Hospital (New Taipei City, Tai-wan) between 2009 and 2016. For OSA, the evaluation involved a comprehensive sleep assessment and evaluation of complaints of excessive daytime sleepiness or snoring; in addition, diagnostic testing and polysomnography (PSG) assessment were performed. OSA was defined as an obstructive respiratory disturbance index (RDI) score ≥ 5 events/h associated with the typical symptoms of OSA (namely, excessive daytime sleepiness, fatigue or insomnia, loud snoring, witnessed apneas, abrupt awakening from sleep accompanied by gasping or choking, awakening with a dry mouth or sore throat, or morning headache), or an obstructive RDI score ≥ 15 events/h (even in the absence of symptoms). In the group that received treatment, 451 patients were treated CPAP, and 171 patients were treated with UPPP. In addition, 106 patients diagnosed with OSA but not on active therapy (NT) were enrolled as the non-exposure group. The exclusion criteria were: age < 35 years, treatment with both CPAP therapy and UPPP surgery, RDI score < 5, previous history of hypertension, and absence of PSG data at baseline (Figure 1). Finally, 196 patients with OSA in the CPAP group, 50 in the UPPP group, and 49 in the NT group were followed up on January 31, 2020.

### Data Collection

All information about the patients with OSA was obtained from the medical charts. The diagnosis codes of OSA were based on the International Classification of Diseases, Ninth Revision, Clinical Modification (ICD-9-CM) codes: 780.57, 780.53, 780.51, 327.23, and ICD-10-CM codes, G47.30, G47.33, and G47.39. Data about the demographic characteristics, comorbidities, index time, treatment, surgery, and PSG re-ports were collected from the medical record system of the hospital. The behavior of CPAP usage had been followed by telephone visits after the patients of the CPAP group were enrolled into this study. The following variables were assessed: age, sex, BMI, occurrence of baseline comorbidities, including diabetes mellitus (DM), chronic kidney disease (CKD), cardiovascular disease (CVD), hyperlipidemia (HLD), and data from PSG reports including Epworth Sleepiness Scale (ESS) score, respiratory disturbance index (RDI), non-rapid eye movement apnea-hypopnea index (NREM-AHI), rapid eye movement AHI (REM-AHI), sleep latency, oxygen desaturation index (ODI), lowest oxygen saturation (LSAT), mean oxygen saturation (Mean SAT), mean heart rate (Mean HR), arousal index, periodic limb movement index (PLMI).

### Study Outcomes

The endpoint of the outcome was presence of hypertension. Blood pressure measured regularly performed at our outpatient department (OPD). Incidental hypertension was confirmed when patients were diagnosed with hypertension at least 2 times and received medicine of blood pressure control for 1 month. The outcome was confirmed by chart review, including OPD note, admission note, emergency room (ER) note, and discharge note. The outcomes were assessed from UPPP or CPAP treatment to onset, the last visit or study's end date (31 January 2020). The onset date of outcome was defined as the first date of the new diagnosis of hypertension or related descriptions. The code of International Classification of Diseases, Clinical Modification, abbreviated ICD-9-CM codes (401.X, 402.X) or ICD-10-CM codes (I10.X, I11. X.) was used to check the visit date and exclusion in those hypertensive patients before UPPP or CPAP treatment. Before the outpatient check of physician, all patients have their blood pressures measured to meet health insurance requirements. For those loss follow-up patients, our follow-up period only counts to the last visit.

### Comorbidities

All the comorbidities, including DM, CVD, CKD, HLD, were clinical diagnosis and confirmed by chart review, including OPD note, admission note, ER note and dis-charge note. The descriptions of participants were reviewed before CPAP, UPPP treatment or first visit in OSA center of NT group. The ICD-9-CM and ICD-10-CM codes were used to track the diagnosis date and the diseases history (Supplementary Table 1).

### Statistical Analysis

The normality assumption not fitted based on Shapiro-Wilk test, so all continuous variables are presented as median (interquartile range, IQR) and tested by Kruskal-Wallis test. Qualitative variables are presented as *N* (%) and were tested using the chi-square test or Fisher's exact test, if necessary.

The date of initiation was considered as the date when the patients started the treatment (CPAP or UPPP) or the patients in the NT group underwent the first PSG test. The end date was the date of onset of hypertension or the last medical visit before the study ended (January 31, 2020), whichever came first. Survival time was calculated from the start to end date and expressed as person-years. The incidence of the out-comes was calculated. Survival curves were estimated using the Kaplan-Meier method and compared using the log-rank test. The Bonferroni method was used for multiple comparisons. The Cox proportional hazards model was used to calculate the unadjusted and adjusted hazard ratios (HRs) and 95% confidence intervals (CIs). The proportional hazards assumption was tested with Schoenfeld residuals; it was not violated in this study.

Inverse probability of treatment weighting (IPTW) was used to balance the distribution of the study population at baseline by weighting the propensity scores of the three treatments. IPTW estimated the average treatment effect by shifting the population from the treated group (CPAP or UPPP) to the NT group (18). One of the ad-vantages of IPTW is that all patients in the study can be used for the outcome evaluation. We did not lose anyone in the analysis. It is no need to sacrifice samples to force the balance sample size (19). A best-fit multinomial logistic model generated the probability of receiving three treatments with adjustments for age, sex, RDI score, index year of enrollment, and comorbidity status of DM, CKD, CVDs, and HLD as weighting variables. The Hosmer–Lemeshow test was used to check the fitness of the logistic models. The weights used in the Cox proportional hazards model obtained a robust non-bias estimation of survival. Statistical significance was set at *P* < 0.05. All statistical analyses were performed using a commercially available software program (SAS version 9.4; SAS Inc., Cary, NC, USA) and R-Studio version 8.10 (R-Tools Technology Inc.; ON, L4C 3C7, CANADA).

## Results

### Participant Characteristics

In this study, 728 patients with OSA were enrolled. Based on inclusion criteria, 196 patients in the CPAP group, 50 in the UPPP group, and 49 in the NT group were included (Figure 1).

The baseline characteristics and comorbidities of all participants are shown in Table 1. Patients in the NT and CPAP groups were significantly older (median age: CPAP vs. UPPP vs. NT: 49.0 vs. 41.5 vs. 50.0, *p* = <0.001), and had a higher incidence of previous CVDs (CPAP vs. UPPP vs. NT: 13.8% vs. 2.9 vs. 18.4%, *p* = 0.019) compared to those in the UPPP group. Several variables were not significantly different between the groups, such as sex, BMI, DM, HLD and CKD (Table 1).

**Table 1:** Baseline characteristics, comorbidities, PSG data of the study participants.

	**CPAP group *N* = 196**	**UPPP group *N* = 50**	**NT group *N* = 49**	***P*** **-value**
**Characteristics**				
Age, yrs, median (IQR)	49.0 (16.0)	41.5 (16.0)	50.0 (13.0)	<0.001
Male sex, *N* (%)	166 (84.7)	54 (77.1)	42 (85.7)	0.306
BMI, kg/m^2^, median (IQR)	28.1 (6.0)	27.2 (3.7)	26.7 (3.9)	0.283
**Comorbidities**				
DM, *N* (%)	18 (9.2)	2 (2.9)	4 (8.2)	0.228
CVD, *N* (%)	27 (13.8)	2 (2.9)	9 (18.4)	0.019
HLD, *N* (%)	27 (13.8)	5 (7.1)	7 (14.3)	0.319
CKD, *N* (%)	1 (0.5)	0 (0.0)	0 (0.0)	1.000
**PSG data**				
ESS, median (IQR)	11.0 (8.0)1	12.0 (6.0)	8.0 (8.0)	0.172
RDI, median (IQR)	48.4 (37.9)	43.8 (36.4)	32.8 (29.2)	0.043
REM AHI, median (IQR)	48.0 (31.2)	46.8 (44.7)	39.5 (29.6)	0.187
NREM AHI, median (IQR)	48.8 (40.1)	43.5 (36.1)	33.6 (29.7)	0.060
Sleep latency, median (IQR)	10.4 (13.6)	17.5 (30.9)	10.0 (17.7)	0.090
ODI, median (IQR)	44.8 (38.2)	42.6 (38.1)	32.4 (28.7)	0.045
LSAT, median (IQR)	77.0 (11.0)	80.0 (11.0)	81.0 (12.0)	0.042
Mean SAT, median (IQR)	94.9 (2.5)	95.4 (1.9)	95.6 (2.3)	0.002
Mean HR, median (IQR)	69.2 (13.1)	68.7 (9.5)	70.3 (13.2)	0.813
Arousal index, median (IQR)	27.6 (31.9)	28.2 (20.6)	28.9 (24.0)	0.366
PLMI, median (IQR)	0.0 (1.1)	0.0 (0.8)	0.0 (4.2)	0.157

*Values are presented as median (IQR), or n (%).

*NT, non-treatment; DM, diabetes mellitus; CVD, cardiovascular disease; HLD, hyperlipidemia; CKD, chronic kidney disease; ESS, Epworth sleepiness scale; RDI, respiratory disturbance index; REM AHI, rapid eye movement apnea-hypopnea index; NREM AHI, non-rapid eye movement apnea-hypopnea index; ODI, oxygen desaturation index; LSAT, lowest oxygen saturation; Mean SAT, mean oxygen saturation; Mean HR, mean heart rate; PLMI, periodic limb movement index; PSG, polysomnography*.

The PSG variables among the three groups showed a similar distribution, except for the RDI, ODI and LSAT. Patients with OSA in the NT group had significantly lower RDI (CPAP vs. UPPP vs. NT: 48.4 vs. 43.8 vs. 32.8, *p* = 0.043) and ODI (CPAP vs. UPPP vs. NT: 44.8 vs. 42.6 vs. 32.4, *p* = 0.045) than patients in the other groups, and the patients in the CPAP group had significantly lower LSAT than patients in the other groups (CPAP vs. UPPP vs. NT: 77.0 vs. 80.0 vs. 81.0, *p* = 0.042) (Table 1).

### Event Rate of Hypertension

In the entire cohort of 295 patients with OSA, the median follow-up period was approximately 3.8 years; there were 43 hypertension events, 1,050.92 person-years accumulated, and the event rate reached 40.95 per 1,000 person-years. Among the three groups, the event rate of incident hypertension was 35.71 in the CPAP group, 45.23 in the UPPP group, and 64.22 per 1,000 person-years in the NT group (Table 2). The comparison of age, gender, BMI, RDI score and comorbidity status (including DM, CKD, CVD and HLD) univariate adjusted model demonstrated in supplementary files (Supplementary Table 2).

**Table 2:** Event rate of hypertension across the three groups.

**Group**	**Median of follow-up year**	**Number**	**Event**	**Person-years**	Event rate^*^
TOTAL	3.8	295	43	1,050.92	40.95
CPAP	4.2	196	27	756.00	35.71
UPPP	3.1	50	7	154.77	45.23
Non-treatment	3.2	49	9	140.15	64.22

*HTN, hypertension; CPAP, continuous positive airway pressure; UPPP, uvulopalatopharyngoplasty*.

* *Event rate per 1,000 person-year*.

### Survival Analysis

**Figure 2:**
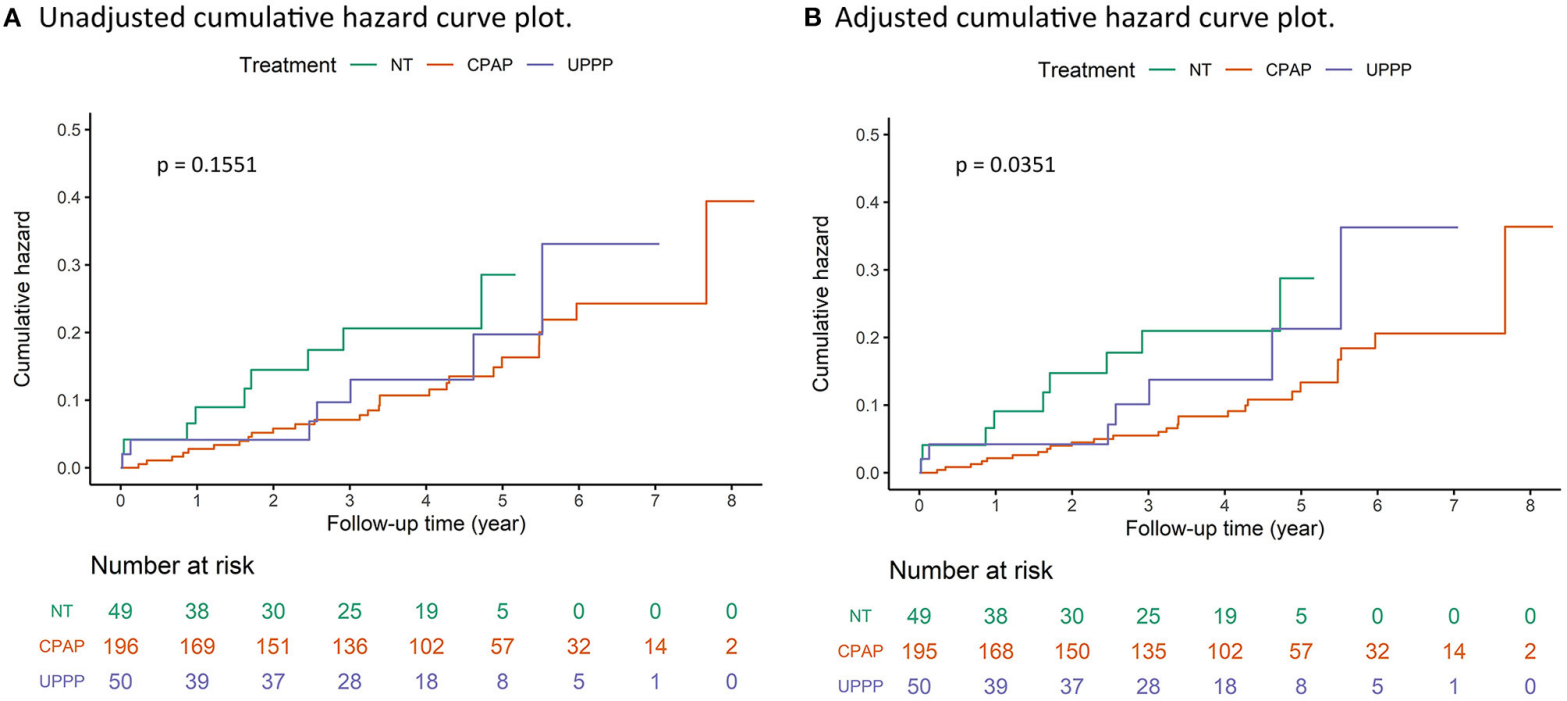
Unadjusted cumulative hazard curve plot and adjusted cumulative hazard curve plot of HTN onset in the CPAP group, UPPP group and NT group, respectively. **(A)** Unadjusted cumulative hazard curves of incident HTN among the three groups were not significant (*p* = 0.1551). **(B)** Adjusted cumulative hazard curves of incident HTN were adjusted by the age, sex, BMI, RDI, and status of comorbidities, including DM, CKD, CVD, HLD. The hazard curves were significant (*p* = 0.0351).

In the Kaplan-Meier analysis, the cumulative survival rates of patients with hypertension were not significantly different between the CPAP, UPPP, and NT groups (log-rank *p* = 0.1551) (Figure 2A). However, after adjustments for the age, sex, BMI, RDI score, status of comorbidities, including DM, CKD, CVD, and HLD, significant differences were found between the three groups (*p* = 0.0351) (Figure 2B).

The risk of hypertension in CPAP group is significantly lower than NT group [HR (95%CI), 0.396 (0.179–0.879), *p* = 0.0227], but UPPP group had no difference with NT group [HR (95%CI), 0.694 (0.248–1.941), *p* = 0.4867].

### Sensitivity Analysis

**Figure 3:**
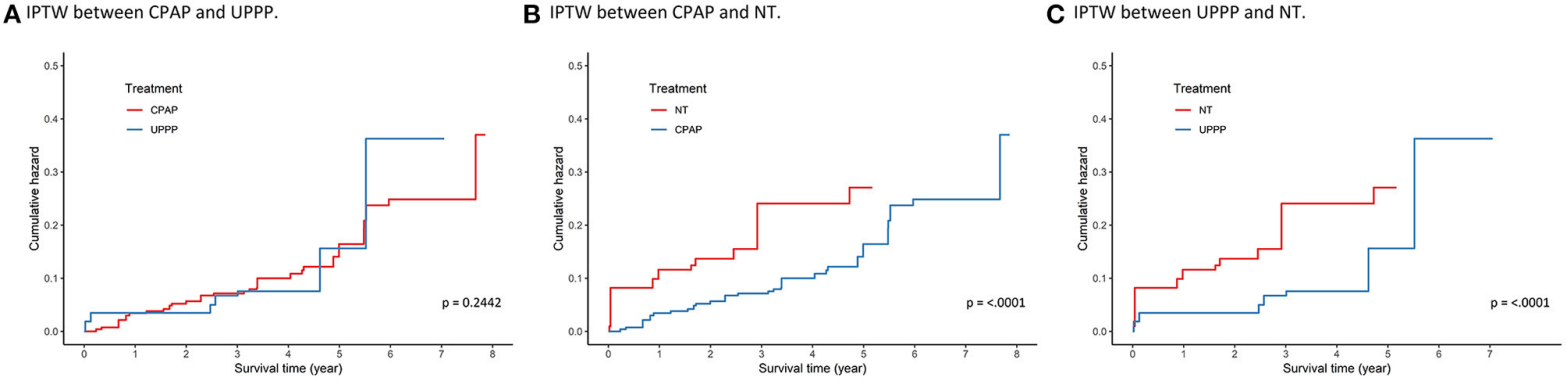
Survival analysis using IPTW between **(A)** CPAP group and UPPP group, **(B)** CPAP group and NT group, and **(C)** UPPP group and NT group. Age, gender, BMI, RDI, index year, and comorbidities status of DM, CKD, CVDs, and HLD were used to calculate the weighting. The Bonferroni significant level is 0.0167. The significances were found in **(B)** and **(C)** (*p* < 0.001). IPTW, inverse probability of the treatment weighting.

IPTW, generated by multinomial logistic regression with the age, sex, BMI, RDI score, index year, and comorbidity status, was used in the Cox proportional hazard model for sensitivity analysis. The results using IPTW in survival analysis were like those of the previous findings in Figure 2. The pairwise comparisons are shown in Figure 3, and the Bonferroni significance level was 0.0167. The patients in the CPAP group had a significantly lower risk of incident hypertension than those in the NT group [HR (95%CI), 0.432 (0.353–0.529), *p* < 0.0001]. Patients with OSA in the UPPP group also had a significantly lower risk of hypertension than those in the NT group [HR (95%CI), 0.414 (0.306–0.560), *p* = < 0.0001]. However, comparison of the patients with OSA in the UPPP and CPAP groups, the HR was not significant [HR (95%CI), 1.162 (0.903–1.495), *p* = 0.2442]. The preventive effect on incident hypertension was similar in both the UPPP and CPAP groups.

In subgroup analysis, we also separate RDI into 2 groups (≥30 vs. <30). Only in those RDI > 30 of OSA patients, CPAP group had lower incidence than NT group (*p* = 0.018) under multivariable adjustments, indicating the preventive effect in onset of hypertension. There was no significant difference in other groups because of small sample size.

## Discussion

This is the first study that showed that UPPP can decrease the incidence of hypertension in patients with OSA. In this study, we found that both CPAP and UPPP had better and more significant preventive effects than no treatment in 4–5 years of follow-up. The preventive impact on the onset of hypertension was seen with both CPAP and UPPP therapy after adjusting for the age, sex, BMI, RDI score, and status for DM, CVDs, and HLD. We also performed IPTW in the survival analysis and found that patients treated with CPAP or UPPP had significantly better prevention than those in the NT group.

In the UPPP group, 42% (*n* = 21) of patients were followed up and had PSG data. Surgical success was commonly defined as <20 RDIs per hour with a drop of more than 50%. In our study, the surgical success rate was 52.4% (*n* = 11), and the improvement rate was even higher. Among the UPPP group, the RDI decreased 27.1/hr in the successes subgroup and 3.06/hr in the non-successes subgroup respectively, therefore, reduced 15.67/hr in total. We expected a success rate of better than 52.4%, first, that patients who responded well to UPPP sometimes lost regular follow-up, and second, that the COVID-19 epidemic in the last 2 years resulted in several intermittent closures of sleep centers. We also reanalyzed the risk of incident hypertension in our study. There were 9% of incident hypertension in successes-in-UPPP patients and 15% in otherwise patients of UPPP group in comparing with the non-treatment group. After multivariate adjustment, 0.38 of hazard ratio in successes-UPPP subgroup to the risk was found but non-significant because of small sample size. The degree of OSA is related to the risk of incidental hypertension. After stratification, we found the incidence of CPAP group lower than NT group when RDI ≥ 30 (*p* = 0.0372)and had no significant difference between groups when RDI < 30 (*p* = 0.4465). The major reason is the study sample size was too small to show the difference.

Several studies have shown that CPAP has a therapeutic effect in reducing blood pressure in hypertensive patients with OSA (10–12, 20). In patients having OSA with hypertension, CPAP led to a significant decrease in systolic blood pressure (SBP) of 2–3 mmHg (11). Furthermore, for patients having OSA with resistant hypertension, CPAP therapy can decrease SBP by 6–7 mmHg. Moreover, there was a significant positive correlation between hours of CPAP use and the reduction in 24-h mean blood pressure (12).

Although secondary prevention of hypertension has been demonstrated in patients with OSA, the primary preventive effect on incident hypertension remains debatable. In 2012, a randomized controlled trial in 14 teaching hospitals in Spain that enrolled 725 patients with OSA showed that CPAP therapy compared with routine care did not result in a statistically significant reduction in the incidence of hypertension (15). Meanwhile, another study of Dr. Marin, which enrolled 1,889 participants without hypertension who were referred to a sleep center in Zaragoza, demonstrated that CPAP therapy was associated with a lower risk of incident hypertension (21). Our findings are like those of Marin's study.

Both studies were performed in Spain, but had different conclusions. There are several reasons for this difference, such as the sample size and study design. However, the most important reason is the difference in the patients' characteristics. In Barbé F's randomized control trial, patients with OSA were non-sleepy, with an ESS score of 6.5 (15). However, the OSA patients in the other study had an ESS score above 10.0. In our study, the ESS score of the participants was 10.0; hence our results were like those of the second study (21). Moreover, the previous two studies included European patients with OSA; however, ours was conducted among Asian patients with OSA. Therefore, we can conclude that CPAP therapy can decrease the incidence of hypertension in European and Asian patients with OSA.

CPAP is the gold standard treatment for OSA; however, the patients' compliance to therapy is challenging. CPAP compliance was defined as CPAP use for at least 4 h per night on 70% of the nights (22). Poor adherence limits the effectiveness of CPAP treatment. Nearly two-thirds of patients with OSA using CPAP experience ad-verse effects, such as air leakage, skin irritation and rashes, nasal congestion, conjunctivitis, claustrophobia, and aerophagy, which contribute to poor adherence to CPAP (23–25). The rate of CPAP non-compliance can range from 29 to 83% (26). In Taiwan, the CPAP acceptance rate was 39.7%, which was significantly low in the elderly group, who had a higher risk of hypertension and cardiovascular disease (27). Besides, aging had a direct correlation with AHI values (28), and the severity of OSA was strongly related to the risk of developing hypertension found by the Wisconsin Sleep Cohort Study (7).

Divide the CPAP group into CPAP-compliance group and CPAP-non-compliance group. The CPAP-compliance group had a lower event rate (33.1 per 1,000 person-year, Supplementary Table 3) of hyper-tension than CPAP-non-compliance group (42.4 per 1,000 person-year, Supplementary Table 3). When we exclude the CPAP-non-compliance patients, the findings are better (Log-rank *p*-value from 0.1551 to 0.0992 in crude analysis and from 0.0351 to 0.0168 in multivariable analysis, Figure 2 and Supplementary Figure 1) among 3 groups (CPAP-compliance group, UPPP group, and NT group). In summary, the results were similar. The patients in the CPAP-compliance group still had a significantly lower risk of incident hypertension than those in the NT group [HR (95%CI), 0.390 (0.305–0.499), *p* < 0.0001, Supplementary Figure 2].

When patients with OSA cannot tolerate CPAP therapy, UPPP is another alternative. Compared with CPAP, UPPP has several advantages, such as requiring only a one-time procedure and leading to less disturbance in life. Regarding hypertension, most UPPP-related articles focused on the therapeutic effect. UPPP has been shown to reduce systemic blood pressure in patients of OSA with hypertension (29, 30). In a study among 65 patients with OSA, significantly reduced blood pressure was observed 24 months after surgery (17). One study showed that UPPP plays an important role in blood pressure control in patients of OSA with hypertension (31). Potential prevention was shown in patients with OSA and hypertension. However, no study has reported the primary preventive effect on hypertension in patients of OSA treated with UPPP.

There is poor evidence about the prevention of incident hypertension in patients with OSA without hypertension. However, some studies have discussed the preventive effect on the onset of cardiovascular disease, including hypertension (32). UPPP can significantly reduce the incidence of complications of hypertension, such as congestive heart failure and atrial fibrillation (33). In 2021, a retrospective cohort study showed that UPPP for patients with OSA was associated with decreased rates of development of cardiovascular complications, including hypertension, myocardial infarction, and coronary artery disease, compared with CPAP therapy (34). In account of our findings, UPPP is one of treatments to prevent hypertension onset in OSA patients.

### Limitation

There are several limitations to this study. First, comorbidities might not have been recorded for some patients with OSA, as they might have received medical treatment at another hospital. To reduce the bias, we arranged telephonic follow-ups for all participants at the end of data collection. Patients who had comorbidities diagnosed at other hospitals but without detailed records were excluded. Second, some data is still lacking, such as gene, income, and personal health insurance data, and the multivariate adjustment of the age, BMI, AHI score, and comorbidities. Third, this was an observational study and not a randomized trial. The unbalanced baseline between the three groups and immortal bias in follow-up is difficult to avoid. However, additional telephonic follow-ups and multivariate Cox models were used to minimize the bias. Since CPAP remains the first choice for patients with OSA, it is difficult to assign patients to different treatments randomly. Fourth, there were 42% (*n* = 21) patients in the surgery group who underwent post-operative assessments, such as the PSG. The success rate of surgery is nearly 52.4% (*n* = 11). The percentage of post-operative follow-up is incomplete because of the COVID-19 pandemic. It is not suitable to represent the total surgery group. Fifth, in addition to UPPP, there are several alternative options for upper airway surgery for OSA, such as maxillomandibular advancement surgery (MMA) (35), selective neurostimulation of the hypoglossal nerve (36) and lateral pharyngoplasty (37, 38), which have higher success rates and lower long-term complications than UPPP (39–41). Besides, lateral pharyngoplasty has effects on blood pressure improvement in patients with obstructive sleep apnea (42).

In this study, we selected UPPP rather than other forms of surgery for two reasons. The first reason is the length of the follow-up period. Information about UPPP treatment for OSA was first published in 1981 (43), but MMA surgery and lateral pharyngoplasty have become popular in recent years. Therefore, the follow-up time of MMA and lateral pharyngoplasty might not be long enough to perform the analysis. The second reason was the sample size. Many surgeons perform UPPP, but only a few doctors in our hospital perform MMA surgery and lateral pharyngoplasty. In the future, we aim to compare different upper airway surgeries for the prevention of hypertension.

## Conclusion

UPPP and CPAP can prevent the development of new-onset hypertension in patients with OSA. CPAP had a better prevention effect than UPPP. Both treatments de-creased the incidence of hypertension in patients aged more than 35 years and had ESS larger than 10.0.

## Data Availability Statement

The original contributions presented in the study are included in the article/Supplementary Material, further inquiries can be directed to the corresponding author.

## Ethics Statement

The studies involving human participants were reviewed and approved by Taipei Medical University, Joint Institutional Review Board. Written informed consent for participation was not required for this study in accordance with the national legislation and the institutional requirements.

## Author Contributions

Y-CL and C-HB: conceptualization, writing—original draft preparation, writing—review and editing, and supervision. Y-CL, C-TC, W-TL, and C-HB: methodology. P-ZC, W-TL, and S-TT: validation. C-HB and S-FL: data curation. Y-CL, C-TC, and P-YC: visualization. C-TC: project administration. All authors have read and agreed to the published version of the manuscript.

## Conflict of Interest

The authors declare that the research was conducted in the absence of any commercial or financial relationships that could be construed as a potential conflict of interest.

## Publisher's Note

All claims expressed in this article are solely those of the authors and do not necessarily represent those of their affiliated organizations, or those of the publisher, the editors and the reviewers. Any product that may be evaluated in this article, or claim that may be made by its manufacturer, is not guaranteed or endorsed by the publisher.

## Abbreviations

BMI: body mass index
CKD: chronic kidney disease
CPAP: continuous positive airway pressure
CVD: cardiovascular disease
DM: diabetes mellitus
ESS: Epworth sleepiness scale
HLD: hyperlipidemia
HTN: hypertension
IPTW: inverse probability of the treatment weighting
LSAT: lowest oxygen saturation
Mean SAT: mean oxygen saturation
Mean HR: mean heart rate
NREM AHI: non-rapid eye movement apnea-hypopnea index
NT: non-treatment
ODI: oxygen desaturation index
OSA: obstructive sleep apnea
PLMI: periodic limb movement index
PSG: polysomnography
RDI: respiratory disturbance index
REM AHI: rapid eye movement apnea-hypopnea index
UPPP: uvulopalatopharyngoplasty.
