# Supplementary material for: Prevention of Incident Hypertension in Patients With Obstructive Sleep Apnea Treated With Uvulopalatopharyngoplasty or Continuous Positive Airway Pressure: A Cohort Study
Source: Front Surg. 2022 Mar 24;9:818591. doi: 10.3389/fsurg.2022.818591 (PMC8987371; doi:10.3389/fsurg.2022.818591)
Supplement: Supplementary file 1 [file Data_Sheet_1.pdf]

Supplementary Material

1 Supplementary Figures and Tables

1.1 Supplementary Figures

Supplementary Figure 1. Unadjusted cumulative hazard curve plot and adjusted cumulative hazard curve plot of HTN onset in the CPAP-compliance group, UPPP group and NT group, respectively.

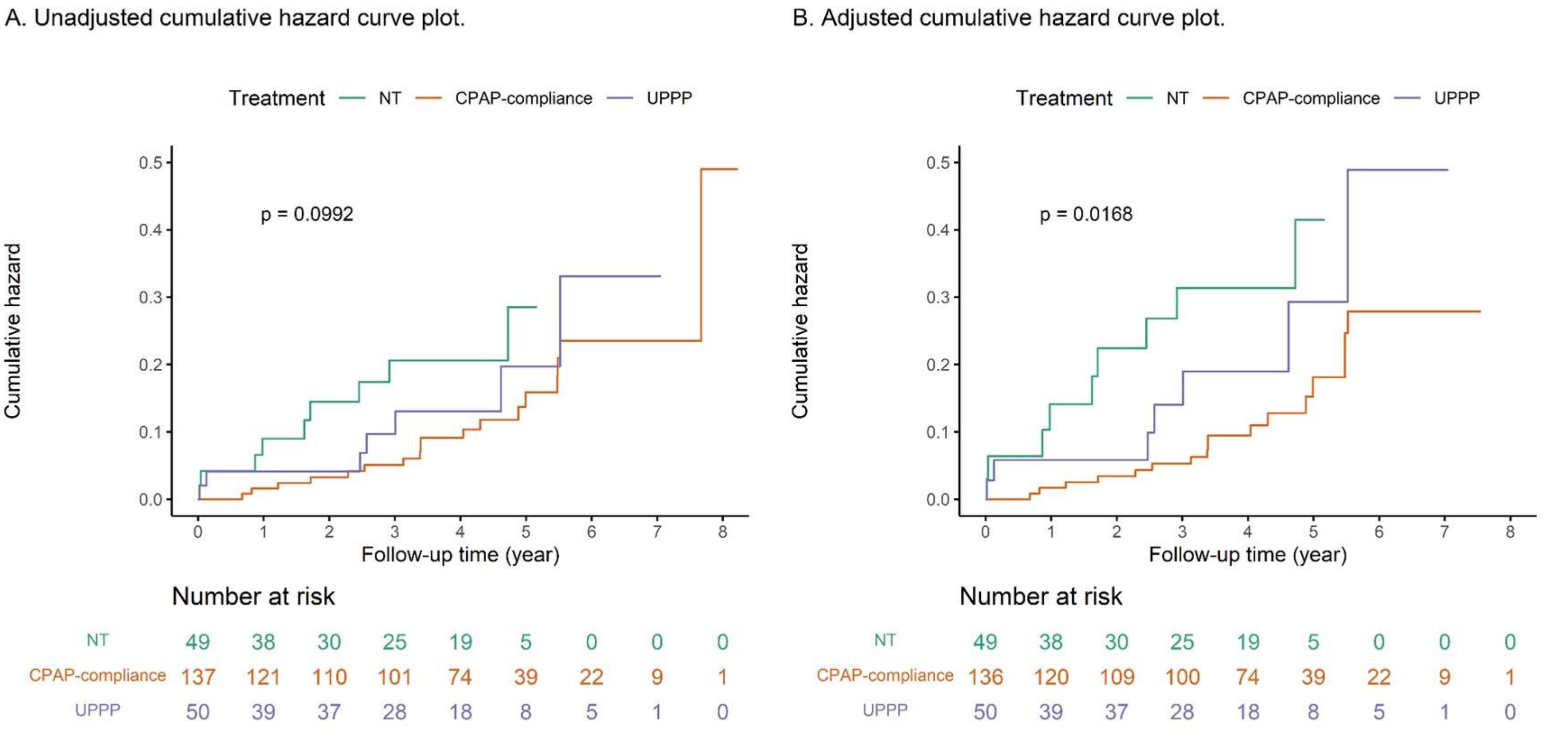

Supplementary Figure 2. Survival analysis using IPTW between (A) CPAP-compliance group and UPPP group, (B) CPAP-compliance group and NT group, (C) UPPP group and non-treatment group.

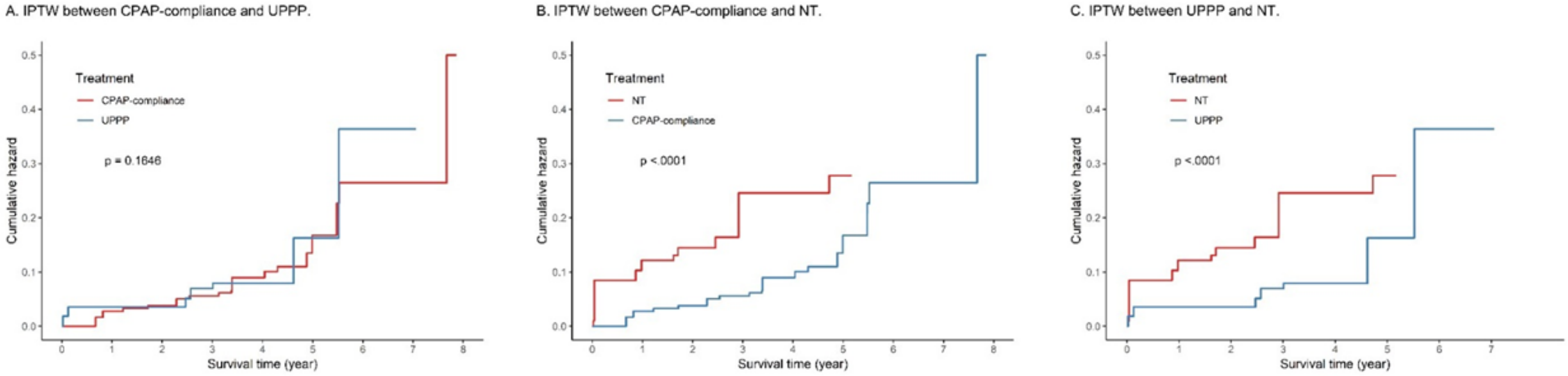

1.2 Supplementary Tables

Supplementary Table 1: ICD-9 and ICD-10 Diagnostic codes used for comorbidities

**Diagnosis**		**ICD-9-CM**	**ICD-10-CM**
**Diabetes mellitus**	*Diabetes mellitus*	250.X	E11.X
**CKD**	*Chronic kidney disease, Hypertensive-chronic kidney disease*	585.X, 403.X	N18.X
**CVDs**	*ischemic heart disease, Angina pectoris, Coronary artery disease, Acute pericarditis, Acute and subacute endocarditis, Acute myocarditis, Other diseases of pericardium, Other diseases of endocardium, Cardiomyopathy, Conduction disorders, Cardiac dysrhythmias, Ill-defined descriptions and complications of heart disease, Subarachnoid hemorrhage, Occlusion and stenosis of precerebral arteries, Transient cerebral ischemia, Atherosclerosis, Aortic aneurysm and dissection, Other aneurysm, Other peripheral vascular disease, Arterial embolism and thrombosis, Atheroembolism, Polyarteritis nodosa and allied conditions, Other disorders of arteries and arterioles, Disease of capillaries, Septic arterial embolism, Phlebitis and thrombophlebitis, Portal vein thrombosis, Other venous embolism and thrombosis, Varicose veins of lower extremities, Other disorders of circulatory system, Symptoms involving cardiovascular system*	411X, 413.X, 414.8-414.9, 420.X-427.X, 429.X, 430.X, 433.X, 435.X, 440.X-449.X, 451.X-454.X, 459.X, 785.X	I20.X, I22.X-I24.X, I25.2-I25.6, I25.8, I25.9, I30.X-I47.X, I51.X, I52.X, I65X, I70.X-I82.X, G45.X, G46.X, M30.X, M31.Xa
**HLD**	*Disorders of lipid metabolism*	272	E78

Supplementary Table 2. Univariable model and multivariable model of survival analysis.

	Univariable model		Multivariable model	
**Variable**	**HR (95%CI)**	**p-value**	**HR (95%CI)**	**p-value**
**Age**	1.010 (0.984-1.038)	0.4520	1.015 (0.982-1.050)	0.3794
**Gender**	0.820 (0.393-1.712)	0.5979	0.671 (0.301-1.492)	0.3273
**BMI**	1.031 (0.967-1.100)	0.3504	1.016 (0.939-1.101)	0.6881
**RDI**	1.010 (0.999-1.022)	0.0753	1.018 (1.004-1.032)	0.0119
**Comorbidities status**				
DM	1.143 (0.444-2.943)	0.7817	0.455 (0.134-1.549)	0.2079
CVD	1.140 (0.507-2.566)	0.7506	1.235 (0.508-3.004)	0.6415
CKD	7.594 (1.033-55.795)	0.0463	40.149 (3.255-495.227)	0.0040
HLD	1.496 (0.715-3.130)	0.2844	1.911 (0.834-4.376)	0.1256

Multivariable models were adjusted by age, gender, BMI, RDI and comorbidities status (DM, CVD, CKD, HLD).

Supplementary Table 3: Event Rate of hypertension among CPAP-compliance / non-compliance group, UPPP group and non-treatment group.

Group	Median of follow-up year	Number	Event	Person-year	Event rate*
TOTAL	3.8	295	43	1,050.92	40.95
CPAP-compliance	4.2	137	18	543.86	33.10
CPAP-non-compliance	3.8	59	9	212.15	42.42
UPPP	3.1	50	7	154.77	45.23
Non-treatment	3.2	49	9	140.15	64.22

*Abbreviations: HTN, hypertension; CPAP, continuous positive airway pressure; UPPP, uvulopalatopharyngoplasty

*Event rate per 1,000 person-years.
